# Influence of aging on opioid dosing for perioperative pain management: a focus on pharmacokinetics

**DOI:** 10.1186/s44158-024-00182-2

**Published:** 2024-08-01

**Authors:** Sebastiano Mercadante

**Affiliations:** Main Regional Center for Pain Relief and Supportive/Palliative Care, La Maddalena Cancer Center, Palermo, 90146 Italy

**Keywords:** Age, Elderly, Opioids, Perioperative pain

## Abstract

The older population continues to grow in all countries, and surgeons are encountering older patients more frequently. The management of postoperative pain in older patients can be a difficult task. Opioids are the mainstay of perioperative pain control. This paper assesses some pharmacokinetic age-related aspects and their relationship with the use of opioids in the perioperative period. Changes in body composition and organ function, and pharmacokinetics in older patients, as well as characteristics of opioids commonly used in the perioperative period are described. Specific problems, dose titration, and patient-controlled analgesia in the elderly are also reviewed. Opioids can be safety used in perioperative period, even in the elderly. The choice of drugs and doses can be individualized according to the surgery, opioid pharmacokinetics, comorbidities, and routes of administration.

## Highlights

• Anesthesiologists are encountering older patients more frequently. The management of postoperative pain in older patients can be a difficult task.

• In the elderly, there are relevant changes in body composition and organ function, as well pharmacokinetics in older patients.

• In this population, opioids, commonly used in the perioperative period, should be carefully used, taking into consideration on pharmacokinetic age-related aspects and available techniques for administering these agents.

## Introduction

The older population continues to grow and lives longer. In the US, about 1/7 of population is older than 65 years, with a 21% increase over the past 10 years [1]. The number of elderly people is expected to double by 2050, reaching a total of 2.1 billion worldwide. Consequently, it is expected a consequent demographic shift toward an elderly population [2]. Of interest, the transition to an older population is observed either in high-income and some middle- and low-income countries [3]. As aged people represent the fastest-growing population of society, this group of people undergoes surgery more frequently than other ones. Thus, surgeons are encountering older patients more frequently, and their surgical demand is expected to increase rapidly.

The rate of morbidity and mortality after surgery increases with age. Postsurgical inhospital mortality has been reported to be significantly lower in patients less than 80 years old than in those greater than 80 years old. Moreover, one-quarter of patients older than 75 years develop postoperative complications [4]. However, although aging is associated with an increase in morbidity and mortality after major surgeries, age alone should not be considered to provide the individual patient’s eligibility for surgery. Elderly individuals are a heterogeneous group. While some of them are highly functional even in their 90 s, others are disabled, frail, or have multiple comorbidities. Emergency surgery, comorbidities, frailty status, severity of illness, and a high American Society of Anesthesia (ASA) score are considered to have a stronger impact on mortality and postoperative complications than age [5]. On the other hand, anesthesiologists are increasingly being involved in the care of frail and elderly patients. The considerable changes in population demographics have been associated with significant improvements in medical technology and knowledge and changes in social norms and health policy. As increasingly complex diagnostic and therapeutic techniques have been introduced, advanced age is no longer seen as a legitimate barrier to surgery and anesthesia.

Postoperative pain in older patients is often underrecognized and undertreated due to lack of a proper pain assessment and concerns of risks of adverse effects [6, 7]. In literature, data regarding the incidence and intensity of postoperative pain in the elderly are conflicting. Some studies suggest that older patients report lower pain intensity than younger ones. While other studies did not report differences. More recent findings have indicated that incidence of postoperative pain moderately decreases with increasing age, with small and questionable clinical significance [8]. Pain management in older patients may be complicated by a number of factors, including a higher risk of age, and disease-related changes in physiology, and disease–drug and drug–drug interactions. Thus, the anesthesiologic management is more challenging. Other than chronic diseases, aging itself increases postoperative mortality and morbidity [9]. Over time, progressive changes in organs and tissues can lead to deterioration of physiological functions [10]. Age-related physiologic changes result in significant changes in pharmacokinetics, that is the relationship between drug dose and plasma concentration, and pharmacodynamics, that is the sensitivity to a given plasma concentration.

The management of perioperative pain in the elderly can be challenging. In a systematic review, significant predictive factors for postoperative pain were preoperative pain, anxiety, age, and type of surgery. In addition, type of surgery, age, and psychological distress were significant predictors for analgesic consumption [11]. Moreover, pain assessment in the elderly may be problematic due to differences in reporting and measurement associated with age-related cognitive impairment. Thus, selection of analgesic management needs to balance the potential efficacy with possible interactions, complications, or adverse effects in the postoperative period [12]. Opioids remain the mainstay of perioperative pain control. Pioneer studies have reported no significant correlation between opioid doses and patient’s weight but a significant correlation with patient age [13–15].

This paper examines some pharmacokinetic age-related aspects and their relationship with the use of opioids in the perioperative period.

### Changes in body composition

With regard to body composition, total body water volume progressively decreases with age. This results in lower volumes of distribution and consequently higher plasma concentrations of hydrophilic drugs [16]. While muscle mass decreases with age, fat forms a relatively greater proportion of the total body mass [17]. Muscle tissues are considered to be “pharmacokinetically active” as they have a high blood flow and participate in distribution of most anesthetic drugs. Indeed, fat tissues have a lower blood flow but a high capacity to slowly absorb drugs, particularly liposoluble drugs. Consequently, volumes of distribution of lipophilic drugs can be similar or even higher in older patients compared with younger ones.

Aging is also associated with changes in the composition of the blood, with lower concentrations of plasma proteins, including albumin or glycoprotein. This phenomenon causes significant changes in the unbound and active fraction of highly protein-bound drugs. In addition, for a significant number of older patients, the use of multiple drugs and coexistent chronic diseases are of concern [18]. A pharmacokinetic model estimated slower rates of fast distribution and a lower capacity for fast distribution in elderly patients [17]. The age-related adjustments in these models are all concordant with the changes in the pharmacokinetics of these drugs in the elderly. When these models are used for controlling target-controlled infusions, older patients receive one or more of smaller induction boluses and lower maintenance infusion rates for the same target concentrations as in younger patients [19].

### Changes in organ function

The function of all organs progressively decreases [20]. Cardiac output is usually modestly affected, but sympathetic activity is reduced so that the response to changes is impaired. Hemodynamic impairment by anesthetic drugs is principally due to a reduced peripheral vascular resistance caused by a decreased vasoconstrictor sympathetic activity. In addition, systemic vascular compliance increases, thus reducing left ventricular preload as well as stroke volume. In patients with good myocardial function, this is accompanied by a counterclockwise shift of the Frank–Starling curve to maintain cardiac output [21]. However, this compensatory activity might be reduced in patients with cardiovascular diseases. Other factors may exacerbate the hypotensive responses to anesthetic drugs and include the use of antihypertensive drugs and diuretics, age-related blunting of *β*-adrenergic responses, a reduced cardiovascular compliance, and comorbidities, such as diabetes and atherosclerosis, which will result in a dysfunction of autonomic reflexes.

Both mechanics and control mechanisms of pulmonary function are affected in the elderly. Chest wall becomes stiffer, and the reduced compliance increases the work of breathing. The decrease of elastic lung activity increases the closing volume. Inspiratory and expiratory functional reserves decline with aging. In addition, protective reflexes are less efficient, and response to hypoxemia and hypercarbia is less effective, so that the use of opioids and benzodiazepines may be of concern [22]. Liver blood flow and size decrease by approximately 35% in aged population. An increasing age and frailty status also impact blood flow and protein binding and drug metabolism. The sum of these deficits with increasing age results in a decline in the functional capacity of the liver. These changes can have a significant influence on the pharmacokinetics and pharmacodynamics of prescribed drugs. Age-related change in liver function causes much of the variability in older people’s responses to drugs [23, 24].

Renal blood flow, glomerular flow rate, and creatinine clearance decrease in parallel with age. This is relevant to drugs that undergo renal clearance. Independently of any comorbid process, both the central and peripheral nervous systems are affected with increasing age due to a loss of cortical gray matter, a decrease of neurotransmitters release and enzymes responsible for postsynaptic degradation, and functional connections decrease. These changes limit the ability to integrate the various neural inputs. Aging is also associated with loss of neuronal activity in the autonomic nervous system [22].

## Pharmacokinetic changes

The pharmacokinetics of most drugs are commonly explained mathematically by a three compartment model, which involves two distribution compartments. In the first compartment (central), the volume into which drugs are initially distributed after intravenous administration is relatively unaffected by age. The other two compartments represent two distribution processes, with two separate distribution processes with different time constants and capacities [17].

The first distribution process is determined by a rapid distribution into a fast distribution compartment, with preferential distribution into well-perfused tissues, such as muscles and organs. For example, for fentanyl, it is the rapid distribution that limits the duration of the clinical effect. In the elderly, rapid distribution is generally slower due to changes in muscle mass and organ size and perfusion. After a loading dose, plasma concentrations and consequently the clinical effect decrease more slowly.

The second distribution process is determined by a slower distribution into a third compartment, that is represented by poorly perfused tissues, such as fat. This process is slow, as these tissues have a high capacity to absorb drugs, especially lipophilic drugs, such as fentanyl or methadone. Considering the body composition changes in older patients, this process may be similar or slower. However, there will be similar or even larger capacity to store drugs, determining a greater tendency to accumulate drugs with lipophilic properties. In noncompartmental pharmacokinetic terms, this will result in a slower elimination half-life. Thus, the overall volume of distribution of lipophilic drugs, which can be represented by the sum of the compartments, can be increased in the elderly [17].

### Metabolism

Glomerular flow rate and creatinine clearance decrease in parallel with age. This is relevant for drugs that undergo renal clearance. Hepatic blood flow declines by 10% per decade of life, and hepatic mass also decreases. Most analgesic drugs are inactivated by hepatic metabolism. The amount of active drug removed from the blood depends on the hepatic blood flow and the hepatic extraction ratio. Extraction ratios depend on lipid solubility and the activity of the metabolizing enzymes. Many analgesic drugs used by elderly patients, unless morphine and hydromorphone, are either cytochrome P450 inhibitors or activators. Metabolic clearance rates exceed hepatic flow rates [25].

### Excretion

Most opioid drugs are excreted by the kidney in both unchanged form of excretion of water-soluble metabolites, resulting from hepatic metabolism of the parent drug (such as morphine 6- and morphine 3-glucuronide, which are the principal metabolites of morphine) [26]. Renal impairment becomes more relevant with increasing age as the glomerular filtration rate declines by an estimated mean of 0.75–0.9 mL/min annually after the age of 30–40 years. Consequently, a patient aged 80 years can be expected to have approximately 2/3 of renal function they had when they were younger [27]. Most opioids are eliminated primarily in urine, making dosage adjustments necessary in patients with renal impairment. Several drugs are excreted changed or unchanged in the bile, including morphine metabolites agents. Thus, age-related reductions in organ blood flow and function result in slower excretion of drugs [28].

## Common opioid analgesics used in the perioperative period

The metabolic pathways for opioids commonly used in the perioperative period are shown in Table 1.

**Table 1:** Metabolic pathways for opioids commonly used in the perioperative period

	CYP2D6	CYP3A3/4	CYP2B6	UGT
Morphine	°°			
Oxycodone	°	°°		
Hydromorphone				°°
Methadone		°°	°	
Tramadol	°°	°°		
Fentanyl		°°		

° or °° means the relevance of CYP on the metabolisms of the various opioids (in column)

### Tramadol

Tramadol is a dual centrally acting analgesic drug with opioid-like effects that acts either by binding to the µ-opioid receptor and inhibiting noradrenaline and serotonin reuptake. Its analgesic potency is 1/3 of that of morphine. Its active metabolite (O-desmethyltramadol) is more potent µ agonist than the parent drug and is renally excreted [29]. With a reduced liver function, metabolic clearance of tramadol is decreased, and consequently, its half-life is increased. Therefore, the dose used in elderly patients with renal or liver failure should be decreased. In older patients, it may be advisable to increase the period between doses. Of interest, 8% of people are resistant to the analgesic effects of tramadol because of a low activity of cytochrome P450 2D6, which converts tramadol to O-desmethyltramadol. Tramadol will result to be ineffective for these patients. Some of its advantages are that it is better tolerated than morphine, with less respiratory depression and constipation [29]. However, the use of tramadol seems to be associated with an increased risk of postoperative delirium [30]. This agent should be used with caution in patients with a history of epilepsy and those taking concomitant seizure threshold-lowering agents [18]. In addition, tramadol has marked interactions with monoamine oxidase inhibitors and with serotonin reuptake inhibitors, often used among older patients. The combination of these drugs with tramadol could expose the patient to serotonin syndrome, which is characterized by autonomic hyperactivity and cognitive-behavioral dysfunction triggered by increased serotonergic stimulation. These phenomena are explained by the reuptake inhibition of serotonin, the release of serotonin in the synapse, and inhibition of monoamine oxidase. For these reasons, the use of tramadol is contraindicated in patients who are receiving MAO inhibitors [31–33].

### Morphine

The physiological changes in elderly patients induce several modifications in morphine pharmacokinetics and pharmacodynamics properties [29]. These include an increase in decrease in volume of distribution, mean elimination half-life, clearance, and protein binding and increase in brain sensitivity. Elimination half-life for morphine is 4.5 h in elderly, significantly longer than the 2.9 h reported in younger patients. After an intravenous morphine injection, the peripheral compartment morphine concentration is higher in the elderly. The increase in analgesic potency of morphine in elderly patients might be due to the increased peripheral compartment concentration and to a longer duration of action. Older patients tend to be more sensitive to equivalent doses and blood levels of opioids, producing greater and more prolonged pain relief [26, 33, 34]. Morphine is highly extracted by the liver and is metabolized into two major metabolites: morphine-3-glucuronide (M3G) and morphine-6-glucuronide (M6G). Hepatic impairment is a nonsignificant factor influencing pharmacokinetics of morphine. M6G accumulates in blood and penetrates the blood brain barrier, binding with strong affinity to opioid receptors and exerting a potent analgesic effect. While M6G is acting as an analgesic, M3G has been shown to antagonize effects of both morphine and M6G or not having any analgesic effect. The parent–metabolite relationship is dramatically changed in patients with renal dysfunction, making opioid toxicity more likely. Physicians should be aware of the risks of administering morphine to patients with severe renal impairment [26, 35]. Evaluation of creatinine clearance is relevant in elderly patients, when using morphine, as the most common adverse effects in older patients with reduced renal function is the risk of respiratory depression. For these reasons, doses of morphine must be reduced by 30–50% in older patients [14, 33, 36].

### Fentanyl-alfentanil

Fentanyl has been the first of a series of opioids characterized by rapid onset, predictable metabolism, and rapid offset. Alfentanil differs from fentanyl for a higher unionized fraction and a smaller volume of distribution, which result in a faster onset of activity. Fentanyl is metabolized in the liver to inactive substances that are excreted in the urine. Less than 10% of the fentanyl is excreted unchanged in the urine. Fentanyl is a high extraction drug, mainly converted by CYP3A4-mediated N-dealkylation to norfentanyl. Fentanyl is bound to plasma proteins. As a consequence, clinical problems affecting protein levels may change plasmatic levels of fentanyl [33]. Fentanyl could be better tolerated than morphine, due to lack of metabolite accumulation. Liver function and use of a CYP3A4 − inducers may significantly change serum fentanyl concentration. Controversies exist about the real influence of renal dysfunction in patients receiving fentanyl. In contrast to morphine, fentanyl is not easily dialyzable. This could potentially be an advantage in some specific conditions. Patients can maintain a relatively constant plasmatic level of fentanyl, without determining a decrease in analgesia due to drug removal during dialysis [26].

Age did not significantly influenced the pharmacokinetics of these drugs [22]. It has been shown that the C50 for EEG depression decreased by 50% in older subjects [37]. This suggests that a pharmacodynamic rather than a pharmacokinetic mechanism may explain the need to reduce the dose in the elderly to approximately 50% to allow older patients to recover from the effects of the drug as quickly as younger patients [38].

### Remifentanyl

The combined pharmacokinetic and pharmacodynamic differences in older patients reduce dose requirements of remifentanil to as little as 1/3 of that required in younger patients. The volume of the central compartment decreases by 20% and clearance by 30 at 80 years. In addition, the C50 for EEG depression is reduced by 50% in elderly subjects. Most importantly, the t½ of plasma-effect site equilibration is also increased. Moreover, an age-dependent increase in the time to maximum drug effect was found. In the elderly, the peak concentration is similar to that of younger individuals after an initial bolus dose, but it takes slightly longer to be reached because of slower equilibration between blood and effect site concentration. The time to peak drug effect is nearly doubled in elderly patients, occurring more than 2 min after a bolus dose is given [39]. This means that blood concentrations are higher in elderly subjects because of a smaller central compartment, although the effect site concentration does not increase because of a less rapid equilibration. The onset and offset are much slower in older subjects [40]. Thus, increased sensitivity and slower onset of action should suggest clinicians to either provide a smaller bolus dose, by 50%, followed by a similar reduction in the infusion rate with adequate allowance for the increased time to effect, before titrating the dose upwards. Alternatively, the bolus dose can be avoided.

Taken together, these data suggest that in the elderly, lower initial doses, infusion rates, and target concentrations of remifentanil should be used. The infusion rates or target concentrations should be increased only after a longer period of time has passed in comparison with younger patients [17].

### Hydromorphone

Hydromorphone is an analog of morphine. Like morphine, it is transformed in the liver by UGT1A3 and UGT2B7 to glucuronides, in three hydrophilic substances eliminated by the kidney. Hydromorphone is not metabolized by CYP450 to any great extent. It has low protein binding (19%) [41]. Hydromorphone and its metabolites accumulate in renal dysfunction, resembling the problems emerging with morphine and its metabolite elimination in such circumstances (the mean ratio of H3G to hydromorphone may increase to 100:1). In fact, these substances are active and may contribute in producing opioid adverse effects. Hydromorphone is 8.5 times as powerful as morphine when administered intravenously [42]. A long-lasting half-life of about 39 h occurs in severe renal impairment as compared to about 15 h in normal renal function [43, 44].

### Oxycodone

Oxycodone is a semisynthetic opioid agonist. Oxycodone is metabolized through CYP3A4 and partially CYP2D6. About 80% is metabolized to noroxycodone formed by an N-demethylation reaction catalyzed by CYP3A4 and 10% to oxymorphone after demethylation by CYP2D6. Noroxycodone, oxymorphone, and conjugated forms of oxycodone are the major metabolites. Oxymorphone contributes minimally with clinical activity of the parent drug.. Genetic changes or drug–drug interactions may easily disrupt this equilibrium, and metabolism diversion may produce unexpected outcomes [45]. Inhibiting both the CYP3A4 and CYP2D6 pathways may change the clearance of oxycodone, highly increasing oxycodone concentrations, and then its toxicity [46]. In different genetic categories grouped as ultrarapid, extensive, and poor metabolizers, different plasma concentrations of the active metabolite oxymorphone were found, although this did not necessarily translate into a clinical difference in oxycodone requirement or analgesic response [47]. Most metabolites, as polar substances produced by hepatic biotransformation, are principally eliminated by kidneys. Clearance of the drug is affected by hepatic and renal dysfunction [26]. Most metabolites are eliminated by the kidneys. Oxycodone has a higher protein affinity (46%) than hydromorphone, suggesting that it may be dialyzed. Clearance of the drug may be affected by hepatic and renal dysfunction [33]. Age is an important factor affecting the pharmacokinetics of oxycodone. Following intravenous administration of oxycodone, patients aged > 70 years are expected to have 40–80% higher exposure to oxycodone than young adult patients [48]. In a two-compartment linear model, lean body mass and age were found to be significant covariates for elimination clearance and the volume of the central compartment. The elimination half-life of oxycodone showed an age-dependent increase [49]. As oxycodone pharmacokinetics are greatly dependent on the age of the patient, it is important to titrate the analgesic dose individually [48]. Dosing should be carefully titrated to avoid considerable accumulation of oxycodone and adverse adverse effects. Plasma concentrations of oxycodone and its metabolites decreased within 240 min of dialysis, causing a little enhance in post-dialytic pain intensity [50].

### Methadone

Methadone is a highly lipophilic substance that is highly protein bound, predominantly a glycoprotein, which is an acute reactive protein fluctuating according to several clinical conditions and concomitant drugs [51]. Higher levels of this glycoprotein may decrease the effects of methadone. Conversely, lower concentrations may increase free plasma concentrations, thereby increasing methadone clinical effects. Accordingly, medications that affect protein binding have the potential to interact with methadone. Methadone has a large volume distribution, with tissue binding being higher than protein binding, and accumulation occurs with repeated dosing. Methadone is a low-extraction drug and is metabolized mainly by N-methylation to inactive metabolites. The activity of CYP3A4 varies considerably between individuals and explains the large differences in methadone clearance and doses needed for analgesia. Methadone is subject to serious drug interactions at CYP3A4. Inducers, inhibitors, or substrates of the CYP450 enzyme system may change methadone metabolism, including antidepressants, antibiotics, antifungals, anticonvulsants and benzodiazepines, cardiac-related medications, and antiretroviral drugs [33]. Some of these drugs are often used in the perioperative period. It does not accumulate in renal failure and is not filtered during hemodialysis, thus maintaining relatively constant concentration [26]. Methadone-induced QT prolongation is of concern, as a large amount of variables may increase its plasma concentration with dramatic consequences, particularly when using high doses. However, the clinical consequences are controversial [52]. Recently, studies have shown that intravenous methadone produces an effective postoperative analgesia and lowers opioid consumption in the postoperative period, without more adverse effects in comparison with other opioid analgesics, and may have an interesting potential to prevent persistent postoperative pain [53]. However, in older patients, the use of methadone may be of concern because of its long half-life and propensity for drug accumulation in older patients.

## Specific problems

Chronic kidney disease is a global health problem, as 50% of the patients with chronic kidney disease are older than 70 years [54]. It is probable, according to information on opioid pharmacokinetics, to propose carefulness with particular opioids in the pain management of patients with chronic kidney disease. Modifications in the dosing or choice of drugs are essential in chronic kidney disease because of complications due to the decreased removal of drugs or their metabolites. Morphine and codeine should be probably avoided in older patients with renal impairment and used with excessive carefulness. Tramadol, oxycodone, and hydromorphone can be used by patient monitoring, while methadone, transdermal fentanyl, and buprenorphine seem to be safe to use in older non-dialysis patients with renal impairment [26]. Clinical suggestions for using and dosing opioids in patients with hepatic or renal impairment are listed in Table 2.

**Table 2:** Clinical suggestions for using and dosing opioids in patients with hepatic or renal impairment

	Hepatic impairment	Renal impairment
Tramadol	Avoid use	Avoid use
Morphine	Reduce dose	Avoid use
Oxycodone	Avoid use	Avoid use
Hydromorphone	Reduce dose	Avoid use
Methadone	Not advised	Reduce dose
Fentanyl	Reduce dose	Recommended

## Dose titration in recovery room

Intravenous administration of opioids is usually recommended for acute pain relief in the immediate postoperative period and the use of small intravenous. Doses of morphine (< 5 mg) allow rapid titration to adequate pain relief. The main challenge is to administer the subsequent doses taking into account the residual effects of the first dose. In a patient who is awake, the presence of pain can be assessed using a verbal or numerical rating scale. For example, an intravenous morphine titration protocol would be when pain increases to 3/10, and intravenous morphine is titrated until pain relief is achieved using a short interval between boluses (5 min) and no upper limit for the total administered dose [55, 56]. A sleepy patient should be considered to have pain relief or morphine-related sedation. Thus, morphine titration is stopped if the patient becomes sedated (Ramsay score > 2), has a ventilatory rate of < 12 bpm, or an oxygen saturation of < 95%, or has other serious adverse effects, such as allergy, hypotension, and severe vomiting. Using this titration protocol, the percentage of patients with pain relief (defined as a pain level ≤ 30 mm) may be up to 98% with a very low incidence of severe adverse events such as severe ventilatory depression (< 1%) [55–57].

The administration of small boluses of morphine probably increases the time to pain relief but decreases the risk of adverse events related to morphine dose accumulation. Repeating i.v. doses of 5 or 10 mg, rather than doses of 2 or 3 mg, is inappropriate in an opioid-naive patient [58–60].

The relationship between the pain relief, as reflected by the pain scores, and time is important. In a large study, patients with higher initial pain scores required more incremental doses of morphine to reach an acceptable level. This relationship may have important consequences. It is important to note that during intravenous morphine titration, pain scores do not markedly change until the morphine dose approaches that dose needed to obtain pain relief and then it abruptly decreases. The use of intravenous titration is aided by an understanding of the time course of pain scores during the pain relief process and the pharmacokinetic properties of i.v. morphine. The administration of small boluses of morphine may probably result in increasing the time to pain relief but decreases the risk of adverse events related to morphine dose accumulation. Repeat intravenous doses of 5 or 10 mg, rather than doses of 2 or 3 mg, is inappropriate in an opioid-naive patient, particularly older patients. Some factors predicting morphine requirements in the recovery room have been examined. Ethnicity (Caucasians), emergency surgery, major surgery, surgery exceeding 100 min, and moderate-to-severe pain were independent predictive factors of early morphine requirements in the the recovery room [61]. In older patients, a reduced dose of opioids is usually recommended because of changes in pharmacokinetics and pharmacodynamics [62]. However, as there is a large variability in dose requirements, titration should adapt the dose to the pain. There is no evidence that titration should take into account the age of the patients. In studies using the same protocol of intravenous morphine titration in young and older patients, pain scores were not significantly different in the two groups before, during, and at the end of morphine titration, and the number of patients with pain relief was also equivalent [55, 58, 63]. In addition, when the dose of titrated morphine was normalized for body weight, that is lower in elderly patients, no significant difference was observed between groups. The number of morphine-related adverse effects, the number of sedated patients, and the number of patients requiring discontinuation of morphine titration were not different. A study after hip surgery confirmed that the dose of morphine (normalized for body weight) was not significantly modified in elderly patients [64]. Thus, the dose of intravenous morphine during “acute” postoperative titration is not significantly modified in elderly patients, although the dose of morphine given over the first 24 h is reduced by 40%. 

## Patient-controlled analgesia

Age-related increases in the analgesic efficacy of morphine have been consistently reported in the postoperative period. In comparison with younger patients, the elderly achieves greater pain relief in response to a fixed dose of morphine [65]. Patient-controlled analgesia (PCA) offers advantages in this population as it provides the opportunity to tailor the doses to the individual. Morphine is the most widely used and presently the most suitable drug for use in PCA in the elderly. Using PCA, patients self-administered less opioid than younger patients, reporting comparable pain relief [14] and high satisfaction [66]. There was an inverse correlation between age and morphine consumption in both males and females, while no correlation was found between morphine consumption and patient weight [64, 67]. Studies have indicated that after acute pain has been brought under control, PCA should be initiated at a dose of 1–1.5 mg, with a lockout period of 5 to 7 min. A continuous background infusion of opioids is not recommended. Morphine is the preferred drug for PCA because it is a well-known, efficient, and inexpensive agent. However, in patients with renal impairment, fentanyl, with no active metabolites, quick onset, and a short duration of action, might be preferred [12, 65]. Education of patients and healthcare professionals is fundamental to optimize the utility of PCA in older patients. Every effort should be made to avoid the development of postoperative confusion, as this is associated with an increased risk of inefficient pain relief and its serious consequences [68]. Close monitoring and evaluation of the patient throughout the perioperative period are required to ensure the appropriate and successful use of PCA in elderly patients. Patients who used opioid-IV PCA were at increased risk of developing delirium compared with patients using postoperative oral opioid analgesics [69]. As neuraxial analgesics may confer equal risk in the development of a postoperative delirium, it seems that factors other than doses of opioid analgesics may play an important role in the development of postoperative cognitive dysfunctions.

## Conclusion

Perioperative pain management of the elderly is complex. Coexistent diseases and concurrent medications put older patients at risk of drug-drug and disease-drug interactions. Selection of appropriate drug therapy requires an understanding of age-related pharmacokinetic and pharmacodynamic changes and should take into account any coexisting diseases and other medications. In the last years, significant improvements in medical knowledge and technology and changes in social norms and health policy have allowed outcomes not imaginable in the elderly until a few years ago. As increasingly complex diagnostic and therapeutic techniques have been introduced, advanced age is no longer seen as a barrier to surgery and anesthesia. Opioids can be chosen according to specific comorbidities and polypharmacy, by using different techniques and routes of administration and advanced monitoring. Research and education about pain management in the elderly should have priority in medicine worldwide.

## Data Availability

No datasets were generated or analysed during the current study.
